# A Rare Overlap: Sjögren’s Syndrome With Autoimmune Hepatitis

**DOI:** 10.7759/cureus.74334

**Published:** 2024-11-24

**Authors:** Sharan Prasaanth, Dhiran Sivasubramanian

**Affiliations:** 1 Internal Medicine, Coimbatore Medical College and Hospital, Coimbatore, IND; 2 Critical Care Medicine, Christian Medical College, Vellore, Vellore, IND

**Keywords:** anti-nuclear antibody, anti-ss-a antibodies, autoimmune hepatitis, autoimmune profiling, chronic liver disease, liver biopsy, overlap syndrome, portal hypertension, salivary gland disorders, sjögren's syndrome

## Abstract

Sjögren's syndrome (SS) is a chronic autoimmune disorder primarily affecting exocrine glands, leading to symptoms such as dry mouth and dry eyes. While SS can occur as a primary condition, it may overlap with other autoimmune diseases, complicating management. Autoimmune hepatitis (AIH), a liver disorder characterized by elevated serum globulins and liver-specific autoantibodies, can co-occur with SS, although this overlap is rare. We report a case of a 45-year-old woman with primary SS who developed abdominal distension, pedal edema, and abnormal liver function tests, raising suspicion of an overlap syndrome. Autoimmune profiling confirmed primary SS, while liver biopsy indicated AIH and chronic liver disease with portal hypertension. The patient received immunosuppressive therapy, including corticosteroids and azathioprine, along with supportive care for cirrhosis led to stabilization. This case highlights the diagnostic challenges and the need for clinical vigilance in identifying autoimmune overlap syndromes to ensure appropriate management.

## Introduction

Sjögren's syndrome (SS) is a chronic autoimmune condition primarily characterized by lymphocytic infiltration of exocrine glands, leading to prominent symptoms such as xerostomia (dry mouth) and xerophthalmia (dry eyes) [1]. This syndrome can occur as a primary condition or secondary to other autoimmune disorders, adding clinical complexity. ​Among the various autoimmune diseases that may coexist with SS, autoimmune hepatitis (AIH) only develops in about 1.7% of patients with SS [2].

AIH is a chronic inflammatory liver disease characterized by autoantibodies and elevated serum globulins, typically affecting women and associated with genetic factors such as HLA-DR3 and HLA-DR4 [3].

SS affects middle-aged women, with a female-to-male ratio of 16:1, and often manifests as a primary condition [4]. This case report presents a 45-year-old female with primary SS alongside features of chronic liver disease. The systemic symptoms suggested a possible overlap with other conditions, leading to a differential diagnosis that initially included Wilson’s disease, non-alcoholic fatty liver disease (NAFLD), and AIH.

Detailed autoimmune profiling, imaging, and biopsies ultimately confirmed the diagnosis of primary SS with associated AIH and chronic liver disease with portal hypertension. This unusual overlap highlights the diagnostic challenges posed by atypical autoimmune disease presentations. Early recognition and treatment are crucial in such cases for preventing disease progression and potential complications, including cirrhosis and hepatocellular carcinoma. This case underscores the need for heightened clinical awareness of rare autoimmune overlaps and careful differential diagnosis to provide appropriate management and improve patient outcomes.

## Case presentation

A 45-year-old female presented with chief complaints of abdominal distension and bilateral pedal edema for the past month. She had complaints of intermittent non-bilious vomiting. She reported a history of progressive breathing difficulty for 10 days and trouble swallowing solid foods. The patient also had complaints of loss of hair and dry skin. The patient reported jaundice one month ago and is a known case of coronary artery disease, post percutaneous coronary intervention of the left anterior descending artery two years back. On examination, the patient had pallor and bilateral pedal edema grade +2. The abdomen was distended and a fluid wave was present. Vitals and ECG were normal. Routine blood investigation showed macrocytic anemia, thrombocytopenia, and elevated inflammatory markers (Table 1). The coagulation profile was normal. The liver function test was abnormal (Table 1).

**Table 1:** Routine blood investigations.

Investigation	Patient value	Reference value
Hemoglobin (Hb)	6.8 g/dl	12-15 g/dL
Mean corpuscular volume (MCV)	110.6 fL	80-100 fL
Platelet count	69,000 cells/μL	150,000-450,000 cells/µL
Total white blood cell count (WBC)	4800 cells/μL	4,500 to 11,000 cells/μL
Serum lactate dehydrogenase (LDH)	747 IU/L	100-225 IU/L
Estimated sedimentation rate (ESR)	60 mm	0-30 mm/hr
C-reactive protein (CRP)	21.6 mg	< 10 mg/L
Total bilirubin	5.6 mg/dl	0.3-1.2 mg/dL
Direct bilirubin	3.3 mg/dl	0-0.3 mg/dL
Serum albumin	2.9 g/dl	3.5-5 g/dL
Alanine transaminase (ALT)	138 IU/L	5-34 IU/L
Aspartate aminotransferase (AST)	74 IU/L	5-35 IU/L
Alkaline phosphatase (ALP)	171 IU/L	40-130 IU/L
Thyroid stimulating hormone (TSH)	4.05 mIU/L	0.4-4.0 mIU/L
Triiodothyronine (T3)	160 ng/dl	80-200 ng/dL
Thyroxine (T4)	13.3 µg/dl	5-12 µg/dL

Ascitic fluid was tapped, and the analysis showed a serum-ascites albumin gradient (SAAG) ratio greater than 1.1 (Table 2), indicating portal hypertension [5]. The culture and sensitivity report showed that the patient was positive for *Klebsiella pneumonia*.

**Table 2:** Ascitic fluid analysis.

Investigation	Patient value	Reference value
Cell count	320 cells/µL	<250 cells/µL
Polymorphonuclear lymphocytes	80%	<25%
Lymphocytes	20%	>50%
Lactate dehydrogenase (LDH)	155 IU/L	<600 IU/L
Albumin	0.2 g/dl	>1 g/dL

The viral panel, rheumatoid factor, and direct Coombs test were negative. Ultrasonography of the abdomen and pelvis showed chronic parenchymal liver disease with mild ascites, and the portal vein diameter was 8.9 mm, with no evidence of thrombosis. Antinuclear antibody (ANA) profiling was done (Table 3), which showed positive antinuclear antibody immunofluorescence with an estimated titer ratio of 1:80 and positive SS-A/RO 60 Kd, SS-A/RO 52 kd antibodies suggesting SS.

**Table 3:** Antinuclear antibody (ANA) profile test. SLE: systemic lupus erythematosus; SS: systemic sclerosis; MCTD: mixed connective tissue disorder

Antibody	Patient result	Associated disease
dsDNA	Negative	SLE
SmD1	Borderline	SLE
Histones	Negative	SLE
SS-A/Ro 60 Kd	Positive	Sjögren’s syndrome
SS-A/Ro 52 Kd	Positive	Sjögren’s syndrome
SS-B/La	Negative	Sjögren’s syndrome
Scl-70	Negative	CREST syndrome/scleroderma/SS
U1-snRNP	Negative	MCTD
Smooth muscle	Negative	Autoimmune hepatitis
LKM1	Negative	Autoimmune hepatitis

**Figure 1:**
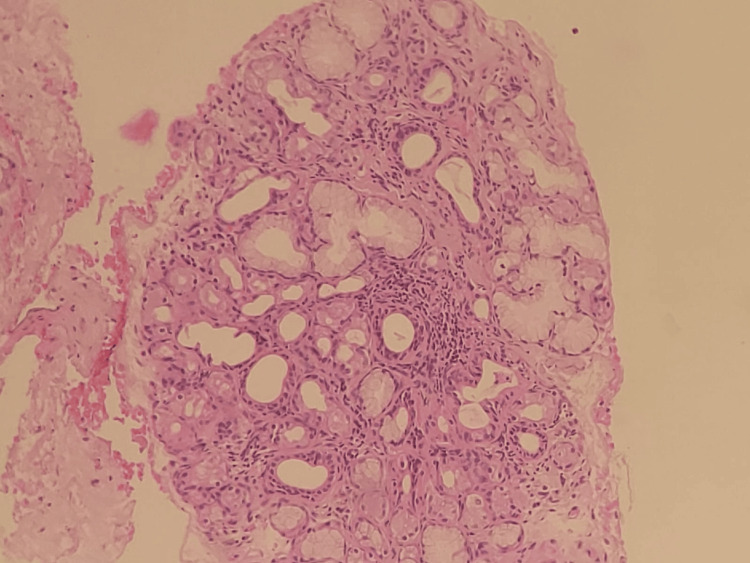
Lip biopsy showed focal lymphocytic sialadenitis suggestive of Sjögren’s syndrome.

The ophthalmic evaluation indicated keratoconjunctivitis sicca with filamentary keratitis. Schirmer’s test was positive. The patient was evaluated for autoimmune hepatitis as serum IgG was mildly elevated. Salivary gland, liver, and lip biopsy were taken. Lip biopsy showed features suggestive of focal lymphocytic sialadenitis pointing toward Sjögren’s (Figure 1).

**Figure 2:**
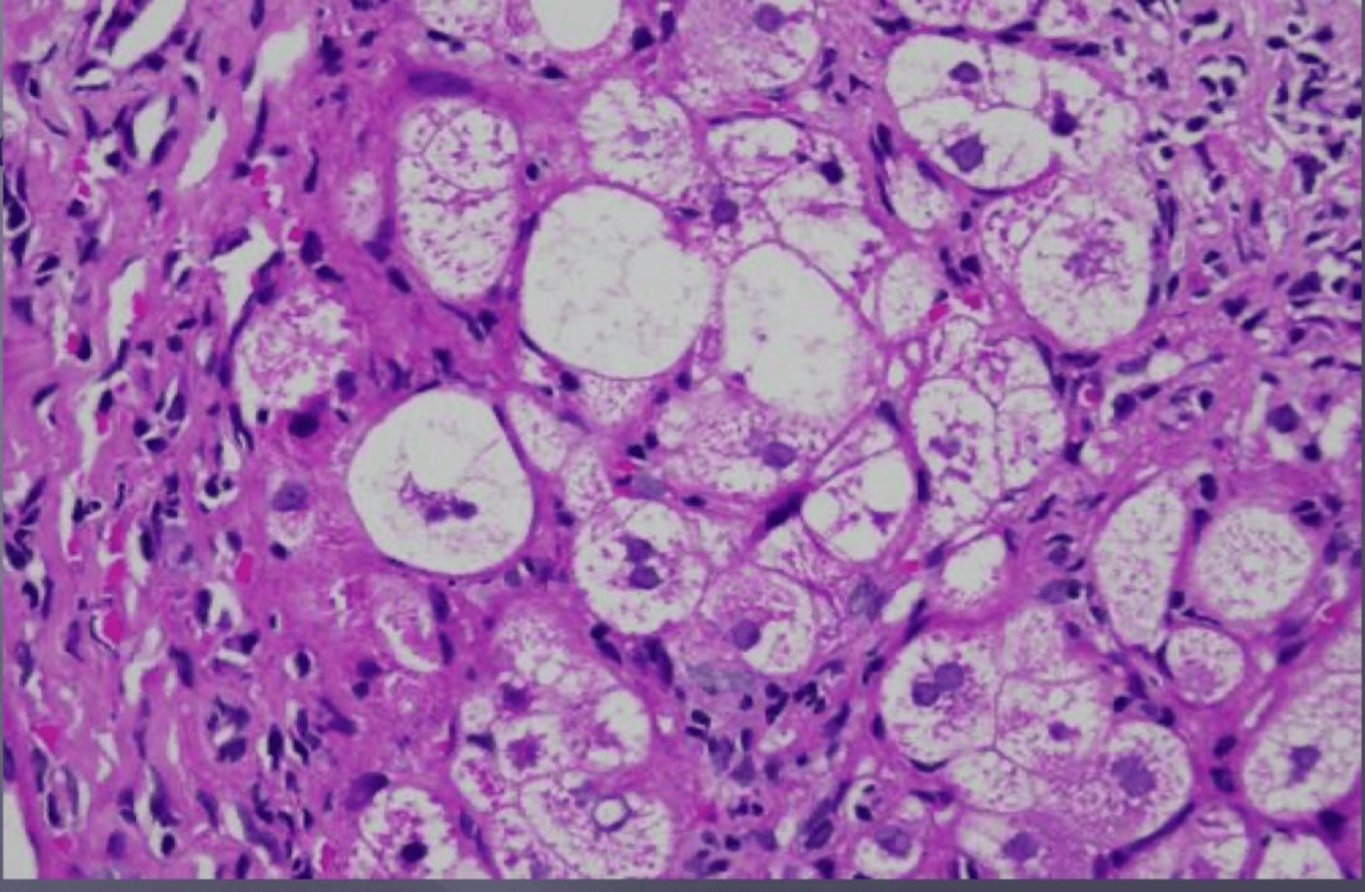
Liver biopsy showed focal lobular inflammation with cytoplasmic features of microvesicular and macrovesicular steatosis and ballooning degeneration indicative of combined chronic non-alcoholic steatohepatitis and autoimmune hepatitis.

A liver biopsy showed lobular inflammation with micro- and macrovascular steatosis and the formation of regenerative nodules suggestive of combined non-alcoholic steatohepatitis and autoimmune hepatitis (Figure 2).

According to the American College of Rheumatology and the European Alliance of Association of Rheumatology (ACR/EULAR) criteria for SS [6] seen in Table 4, the patient had a score of 7, which is above the four points required for the diagnosis of SS.

**Table 4:** Diagnostic criteria for Sjögren's syndrome according to the American College of Rheumatology and the European Alliance of Association of Rheumatology (ACR/EULAR) Patients with a score of ≥4 are classified as primary Sjögren’s syndrome. Reference: [6]

Clinical feature	Points
Focal lymphocytic sialadenitis	3
Anti-SSA positive	3
Ocular staining score (OSS)	1
Schirmer’s test ≤ 5 mm/5 minutes in at least one eye	1
Unstimulated whole saliva flow rate ≤0.1 ml/minute	1

The patient had a score of 6 in the AIH diagnostic criteria established by the International Autoimmune Hepatitis Group in 2008 [7], seen in Table 5, which required a score of 6 or more to diagnose with probable AIH.

**Table 5:** Diagnostic criteria for autoimmune hepatitis (AIH) established by the International Autoimmune Hepatitis Group in 2008 [7]. An aggregate score without treatment ≥7 is definite autoimmune hepatitis (AIH) while ≥6 is probable AIH.

Clinical feature	Points
Autoantibodies	
ANA or SMA ≥ 1:40	+1
ANA or SMA ≥ 1:80 or LKM1 ≥ 1:40	+2
SLA-positive	+2
Serum IgG	
>upper limit of normal	+1
>1.1 times upper limit of normal	+2
Histologic findings	
Compatible with AIH	+1
Typical of AIH	+2
Hepatitis viral markers	
Negative	+2

The patient was managed with a combination of supportive and targeted therapies. To treat the *Klebsiella pneumoniae* infection identified in the ascitic fluid culture, intravenous piperacillin-tazobactam was administered. Immunosuppressive therapy with corticosteroids and azathioprine was initiated to address the autoimmune components associated with SS and probable autoimmune hepatitis. For liver disease management, diuretics furosemide and spironolactone were added to control fluid overload related to cirrhosis, and ursodeoxycholic acid was given to support liver function. In addition, rifaximin and lactulose syrup were prescribed to prevent hepatic encephalopathy.

The patient’s anemia was managed with a transfusion of one unit of packed red blood cells. Nonsteroidal anti-inflammatory drugs (NSAID) and statins were administered as needed for symptom control, and dietary modifications, including salt and fluid restriction, were implemented to support fluid balance. For SS, artificial tears and saliva substitutes were recommended to alleviate eye and mouth dryness. The patient was also advised on lifestyle modifications to avoid factors that could exacerbate dryness and received guidance on the importance of regular dental check-ups to prevent complications associated with reduced saliva production.

## Discussion

SS is a chronic autoimmune disorder predominantly affecting women between the ages of 50 and 60, with a striking female-to-male ratio that suggests a genetic predisposition [8]. An X-linked gene may contribute to this gender disparity, alongside associations with the HLA-DR3 and HLA-DR4 genes [9]. A hallmark of SS is lymphocytic infiltration in exocrine glands, resulting in xerostomia and keratoconjunctivitis sicca [1]. Beyond these classic manifestations, SS can also present with extra-glandular features, including dental caries, gastroesophageal reflux disease, pneumonitis, and even lymphoma, which complicates the clinical picture [10].

The underlying pathophysiology of SS involves CD4+ T cells and B cells infiltrating the exocrine glands, and a positive rheumatoid factor autoantibody and ANA are found in 50-80% of cases [8]. Two key ribonucleoprotein antigens, SS-A (Ro) and SS-B (La), are also often present [8], as seen in this patient with a positive SS-A/Ro antibody. A proposed hypothesis suggests that a viral infection may trigger aberrant T and B cell activation, leading to autoimmune damage in glandular tissue [11]. Anti-SSA Ro antibodies, positive Schirmer’s test, and focal lymphocytic sialadenitis on lip biopsy provided sufficient criteria, with a score of 7, to diagnose primary SS in this patient.

AIH, although rare in SS, presents a unique clinical challenge when overlapping. AIH is a chronic inflammatory liver disease with variable presentation, ranging from non-specific symptoms like fatigue and nausea to fulminant hepatitis. The disease has a five-year mortality rate of around 50% if left untreated, making early intervention crucial [12]. AIH pathogenesis also involves CD4+ and CD8+ T lymphocytes and, like SS, has associations with HLA-DR3 and HLA-DR4 genes [13]. Triggers for AIH may include viral infections, drugs, or even vaccines. AIH typically involves type 1 AIH markers such as antinuclear antibodies (ANA), smooth muscle antibodies, anti-actin antibodies, and P-ANCA [10]. The absence of systemic infectious signs, such as fever or leukocytosis, along with the patient's history of chronic symptoms rather than acute manifestations commonly seen in infections, rules out infection as the root cause of her presentations.

Diagnosing an overlap syndrome, like SS and AIH, requires careful consideration of clinical, serological, and histological analysis. In this patient, elevated liver enzymes, abnormal liver biopsy findings including micro and macrovascular steatosis with ballooning degeneration and regenerative nodules, negative viral panel, and ANA titer >1:80 gave an AIH score of 6, which supported the diagnosis of AIH. The management of such patients involves corticosteroids and azathioprine, which help to control the immune-mediated damage to both exocrine glands and liver tissue [10]. Corticosteroids, however, may increase the risk of candidiasis and dental caries in SS patients. The addition of artificial tears, saliva substitutes, and lifestyle modifications provided symptom relief for SS-related dryness [10].

The overlap between SS and AIH is rare, with an estimated prevalence of less than 1% [2]. Yet, such cases highlight the importance of recognizing autoimmune overlap syndromes, as they can lead to severe complications such as cirrhosis and hepatocellular carcinoma. Since the first known report by Christiansson et al. [14], the coexistence of SS and AIH has intrigued researchers, with some studies suggesting that SS may increase susceptibility to liver involvement. This case emphasizes the need for heightened clinical awareness, thorough autoimmune profiling, and histopathological evaluation in patients with SS presenting with abnormal liver function.

## Conclusions

This case underscores the importance of a comprehensive diagnostic approach, including detailed autoimmune profiling and histopathology, to exclude other autoimmune disorders and provide accurate diagnoses in complex overlap syndromes. Effective management requires a multidisciplinary approach, using a combination of immunosuppressive therapy and supportive care tailored to each condition. Further studies are needed to develop standardized guidelines for managing patients with SS-AIH overlap, as early recognition and intervention are critical to prevent progression to cirrhosis and hepatocellular carcinoma. This case contributes to the understanding of autoimmune overlap syndromes and emphasizes the need for research to improve therapeutic interventions for autoimmune overlap syndromes.
